# Study of antioxidant activity during the malting and brewing process

**DOI:** 10.1007/s13197-019-03851-1

**Published:** 2019-06-20

**Authors:** Dániel Koren, Szilárd Kun, Beáta Hegyesné Vecseri, Gabriella Kun-Farkas

**Affiliations:** Department of Brewing and Distilling, Faculty of Food Science, Szent István University, 45 Ménesi Street, Budapest, 1118 Hungary

**Keywords:** Beer, Malts, Brewing, Malting, Antioxidant activity

## Abstract

In this study the evolution of antioxidant activity was investigated during malting of different barley cultivars, and during the production of different types of beers on laboratory scale and in pilot brewery. Samples were taken at technologically important points of productions. Malts were produced from 3 spring and 3 winter barley cultivars. Two types of beers were brewed under laboratory conditions, and two in a pilot brewery. For the determination of antioxidant activity five commonly used assays were applied such as ABTS Radical Scavenging Activity, Cupric Reducing Antioxidant Capacity, DPPH Radical Scavenging Activity, Ferric Reducing Antioxidant Power and Total Polyphenol Content. Prior to malting it was observed that there are orders of magnitude differences between the antioxidant activities of the barley varieties. During malting, the biggest increase was noticed during steeping. Spring and winter cultivars showed similar trends during steeping and germination, but kilning had different effect on antioxidant activity of the varieties. The antioxidant activity of malts was always higher than the corresponding barleys. During the brewing process antioxidants were released to the highest extent during the early stages of mashing. Adequate sparging and hop boiling could further improve the antioxidant potential of the wort. Furthermore, differences between the equipment used for wort separation and hop boiling under laboratory conditions and in the pilot brewery had effect on antioxidant activity. In the course of malting and brewing by selecting the appropriate raw materials and technological parameters, the conditions for the release and retention of antioxidants can be optimized.

## Introduction

Technological steps of malting and brewing do have a significant effect on the composition of malts and beer made of them. These steps not only influence the extract, alcohol or protein content of the final product but the bioactive components like antioxidants, as well. This study focuses on the evolution of antioxidant activity during malting of different barley cultivars, and during the production of beers made of different types of malts in laboratory and in pilot brewery.

### Antioxidants

Antioxidants are important compounds which help us to retain our health. The main role of antioxidants in human health is to attenuate oxidative stress. Oxidative stress arises from overproduction of reactive oxygen or nitrogen species (ROS/RNS). These free radicals are produced under normal physiological and pathological conditions in our organism and play an important role in pathological processes and regulatory activities. Antioxidants can act in different ways, they can scavenge free radicals, inhibit pro-oxidative enzymes, chelate metal ions, among others. Grains are excellent sources of antioxidants, such as vitamin E, polyphenols, phytic acid, folates and microelements (e.g. zinc, selenium) (Shahidi et al. 2012).

### Determination of antioxidant activity

According to Huang et al. (2005) there are two categories of assays which are applicable to determine antioxidant activity, these categories are hydrogen atom transfer reaction-based assays and single electron transfer reaction-based assays. In this study electron transfer based assays were applied. These methods involve two components in the reaction mixture, antioxidants and oxidant. They are based on a reaction in which the probe (oxidant) is reduced by the antioxidant (present in the sample) that cause color change which can be measured by a spectrophotometer. The degree of the color change is proportional to the antioxidant concentrations. The reducing capacity of the antioxidant is usually expressed as Ascorbic acid equivalent (AAE), Trolox equivalent (TE) or gallic acid equivalent (GAE). These assays have the limitation that they are not selective to certain compounds, only suitable for determining the reducing capacity of the sample. On the other hand, significant correlation can be found between antioxidant activity and certain groups of antioxidants, e.g. polyphenol content in beer (Zhao et al. 2010).

### Antioxidant potential of barley and malt

Malted barley is used in the second highest proportion after water in brewing. The most significant antioxidants in barley are polyphenols. Many scientific papers have reported that the long-term consumption of plants rich in polyphenols protects us against development of cancers, cardiovascular diseases, osteoporosis, diabetes and neurodegenerative diseases (Graf et al. 2005). Phenolic substances are an important part of the natural defense system of plants, protecting them from bacterial and fungal pathogens, insects and herbivores (Zimmermann and Galensa 2007). The phenolic content of barley is influenced by biotic and abiotic factors, which affect plant physiology and secondary metabolites (Mikkelsen et al. 2015). Phenolic compounds are predominantly found in the outer layers of the grain (husk, pericarp, testa, aleurone cells) bound to cell wall polysaccharides (Naczk and Shahidi 2004; Kähkönen et al. 1999). During malting, the extractability of these compounds is increasing mainly due to enzymatic processes and better friability.

The other group of potential antioxidant compounds is developed during the heat treatment of green malt. In case of special malts (e.g. caramel, coloring) which are treated at high temperature (150–200 °C) at the end of the malting process, Maillard reaction takes place intensively resulting in products that do have antioxidant activity (Carvalho et al. 2015).

### Antioxidant potential of hops

Hops, the other important ingredient of beer regarding to antioxidants, contain higher concentrations of polyphenols than barley malt (barley malt 50–100 mg/100 g, hops up to 4 g/100 g), but barley malt contributes 70–80% of total polyphenols in traditional beers (Almaguer et al. 2014; Narziss 1976).

### Effect of technological steps during brewing on antioxidants

There have been studies investigating antioxidants from many aspects related to brewing. Schwarz, Boitz and Methner (2012) studied how the mashing-in temperature influences the release of polyphenols. They found that 40–45 °C is ideal for phenolic acid release from malt, while at temperatures above 65 °C no enzyme activity related to release of phenolic acids was detected. Fumi et al. (2011) studied polyphenols in all-malt worts and in maize adjunct worts, and their fate during the main brewing steps. They observed higher phenolic content in all-malt worts than in worts with maize adjunct, furthermore they reported that the overall brewing process reduces by 50% the initial content of total phenols. Zhao (2015) investigated the effects of processing stages on the profile of phenolic compounds from barley to the final product. It was found that their amount had generally increased significantly during malting and mashing but decreased markedly during the subsequent fermentation and storage. Pascoe, Ames and Chandra (2003) studied the effect of critical stages of the brewing process on antioxidant activity. They observed a decrease after beer filtration, and increase in levels of antioxidant activity after mashing, boiling, fermentation and chill-lagering.

The number of studies focusing on antioxidants related to brewing have increased in recent years proving the importance of this topic. With this study we would like to widen knowledge on the subject by comparing the antioxidant activity of spring and winter barley cultivars, and its evolution during the malting process. Furthermore, antioxidant activity during the whole brewing process of different beer types, produced using both basic and special malts, under laboratory conditions and in pilot brewery have not been investigated and compared yet.

## Materials and methods

### Barley samples

Six barley cultivars were involved in the study. Malts were produced from all of them. Among them there were 3 spring barley cultivars: Quench, Malz and Kangoo, and 3 winter barley cultivars: Casanova, Vanessa and Wintmalt.

### Malting

The malting was carried out from the above-mentioned barley cultivars in a Schmidt-Seeger micromalting plant (Bühler AG). Samples were taken and antioxidant activity was determined from barley before malting, during malting after steeping, every day during germination and after kilning.

The malting technology was as follows: steeping consisted of 3 sessions, each session lasted for 8 h (4 h of wet period and 4 h of air rest) at 16 °C. The germination lasted for 4 days, the temperature was 18 °C on the first day, 20 °C on the second day, 22 °C on the third day and 20 °C on the fourth day. Water was sprayed 5 times per hour onto the green malt during germination, and 30 rotations were set in every 2 h. The kilning began at 40 °C, the temperature was raised to 48 °C in 16 h, then raised to 67 °C in 4 h, afterwards to 83 °C in 2 h, and finally cooled to 30 °C in 10 h.

### Brewing

Four types of beers were brewed, two under laboratory conditions and two in a 50-liter capacity pilot brewery.

#### Beers brewed in laboratory

In laboratory two types of lagers (Pilsner and Vienna lager) were brewed. Samples were taken at the end of all enzymatic rests during mashing, after wort separation, after hop boiling, on every day of the main fermentation, and at every third day of the chill-rest. The mashing of beers produced was carried out in a 1-CUBE mashing bath (1-CUBE s.r.o., Czech Republic).

The recipe of the Pilsner type beer was as follows: the water: malt ratio was 4:1. 100% Pilsner malt was used. Hops: Magnum (15% α-acid). Yeast: Saflager W34/70 (Fermentis).

The recipe of the Vienna lager was the same as Pilsner beer except for the malt composition. For the Vienna lager 1:1 Pilsner: Vienna malts were used.

The brewing technology of beers brewed under laboratory conditions was as follows: mashing-in was carried out at 52 °C, then the temperature of the mash was held at 52 °C for 20 min, that was followed by a 45 min rest at 63 °C, and finally there was a 15 min rest at 73 °C. The temperature between the enzymatic rests was raised by 1 °C/min. The wort separation was carried out using Whatman MN-615 filter paper (GE Healthcare). The hop boiling lasted for 60 min in Erlenmeyer flask, at the 5th min 0.8 g Magnum hops was added. The wort was cooled to 12 °C prior to fermentation. The main fermentation lasted for 6 days at 12 °C followed by a chill-rest for 15 days at 5 °C.

#### Beers brewed in pilot brewery

In the 50 L capacity pilot brewery two types of ales—Brown ale and Stout—were produced. Samples were taken at the end of all enzymatic rests during mashing, after first wort separation, after sparging (from the sweet wort), after hop boiling and at every day of the main fermentation.

The recipe of the Brown ale was as follows: the water: malt ratio was 4:1. The malt composition was 60% Maris Otter pale, 15% Vienna, 10% Carapils, 10% Cara hell and 5% Chateau Special B. Hops: Challenger (8% α-acid), yeast: Safale S-04 (Fermentis).

The brewing technology of the Brown ale was as follows: mashing in was carried out at 52 °C, then the temperature was held for 20 min at 52 °C, then it was increased to 63 °C and held for 45 min, then increased to 73 °C and held for 15 min, finally the temperature was raised to 78 °C prior to mashing out. The temperature between the enzymatic rests was increased by 1 °C/min. The mash was pumped into the lauter tun which was followed by a 20 min sedimentation rest. Then the first wort was separated and was followed by two times sparging. The hop boiling of the sweet wort lasted for 90 min, at the 5th min 36 g Challenger hops were added. Then the hopped wort was pumped into the whirlpool where the hot trub was separated. The hopped wort was cooled to 21 °C prior to fermentation. The main fermentation lasted for 4 days at 21 °C.

The recipe of the Stout was as follows: the water: malt ratio was 4:1. The malt composition was 45% Maris Otter pale, 40% Smoked pale, 10% Cara Bohemian, 2% Carafa III. and 3% Chocolate malt. Hops: Warrior (17% α-acid), yeast: Safale S-04 (Fermentis).

The brewing technology of the Stout was as follows: mashing in was carried out at 45 °C followed by a 15 min rest at 45 °C, then the temperature was increased to 55 °C and was held for 15 min, it was followed by a 45 min rest at 63 °C, then the temperature was raised to 73 °C and held for 15 min, finally the mashing out was carried out at 78 °C. The mash was pumped into the lauter tun and was followed by a 20 min sedimentation rest. Then the first wort was separated and was followed by three times sparging. The hop boiling of the sweet wort lasted for 60 min, at the 5th min 20 g Warrior hops was added. The whirlpool, cooling and fermentation was the same as in case of Brown ale.

### Sample preparation and extraction

The extraction of barley and malt samples for antioxidant capacity determination was as follows: for 2 g of finely ground sample, ground in an EBC mill, 20 mL of 80:20 Acetone: Distilled water solution was added. It was sonicated for 10 min in an ultrasonic bath, shaken for 60 min at 150 rpm and centrifuged at 2500 g for 10 min. The supernatant was collected and stored at − 80 °C until analysis.

### Analyses of antioxidant activity

The antioxidant activity was determined by five commonly applied assays as there is no standard method which can objectively characterize this parameter. The following assays were applied because these are widely used to determine this parameter so there is a possibility to compare our results with others’, furthermore these assays are easily reproducible, however are not selective to certain components, these methods are determining the reducing ability of the sample. (Huang et al. 2005) All the results were expressed as mg/100 g or mg/100cm^3^ ascorbic acid equivalent (AAE), in case of malts related to dry matter (d.m.). All the measurements were carried out in three parallels.

#### ABTS radical scavenging activity

The assay was performed as described by Re et al. (1999). 10 µL degassed sample was pipetted into 96 well plates. 20 µL solution was added, which contained 9% NaCl, 1% glucose, 50 mg/mL myoglobin dissolved in pH 7.4 potassium-phosphate buffer. Then 150 µL 1 mg/mL 2,2′-Azino-bis (3-ethylbenzothiazoline-6-sulfonic acid) diammonium salt (ABTS) solution and 25 µL 3% H_2_O_2_ dissolved in 0.1 M pH 5 citric buffer was added. It was shaken for 15 min at 37 °C then absorbance was measured at λ = 405 nm.

#### Ferric reducing antioxidant power

The assay was performed according to Benzie and Strain (1996). Samples were added to FRAP reagent that contained 10 mM 2,4,6-Tris(2-pyridyl)-s-triazine (TPTZ) dissolved in 40 mM HCl, 300 mM pH 3.6 acetate buffer and 20 mM FeCl_3_*6H_2_O. After 5 min of incubation time, absorbance was measured at λ = 593 nm and.

#### Total polyphenol content

The assay was performed based on the description of Singleton and Rossi (1965), which is based on the reduction power of antioxidants rather than on the selective reaction of polyphenols, thus it was evaluated together with the other antioxidant activity assays (Martinez-Periñan et al. 2011). First 1250 µL ten-fold diluted Folin-Cioalteau reagent and 240 µL methanol: water (4:1) solvent were pipetted in the test-tubes. Then 10 µL degassed sample was added. After homogenization and 1 min reaction time 1 cm^3^ 0.7 M Na_2_CO_3_ was added, vortexed and before measurement the mixture was allowed to stand for 5 min at 50 °C. The absorbance was measured at λ = 765 nm.

#### Cupric reducing antioxidant capacity

The assay was performed according to Apak et al. (2004). 100 µL sample was added to 1 cm^3^ 10^−2^M CuCl_2_, 1 cm^3^ 7.5*10^−3^M neocuproine solution (dissolved in 96% ethanol), 1 cm^3^ pH 7.4 1 M NH_4_Ac buffer solution and 0.9 cm^3^ distilled water. It was incubated in dark for 30 min and the absorbance was measured at λ = 450 nm.

#### DPPH radical scavenging activity

The assay was performed as described by Brand-Williams, Cuvelier and Berset (1995). 6*10^−5^M 2,2-Diphenyl-1-picrylhydrazyl (DPPH) solution was prepared with methanol. 100 µL sample was added to 3.9 cm^3^ DPPH solution and was incubated in dark for 20 min then the absorbance was measured at λ = 517 nm.

### Real extract content

The real extract content was determined by an Anton-Paar Alcolyzer Plus beer analyzer.

### Moisture content

The moisture content of the barley and malt samples was determined by an AND MX-50 Moisture Analyzer.

## Results and discussion

### Antioxidant activity of barleys and malts

**Fig. 1:**
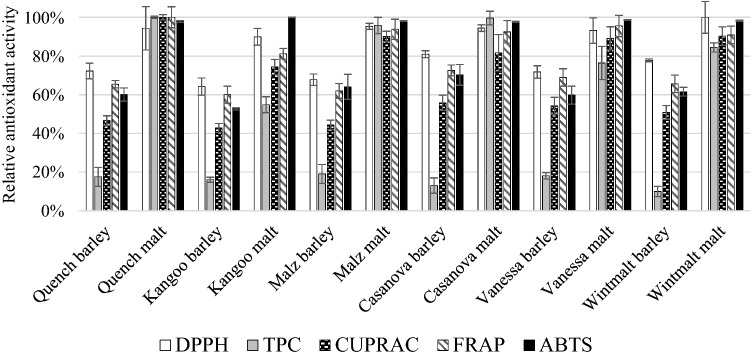
Relative antioxidant activity of barleys and malts (n = 3, mean, sd). DPPH, DPPH Radical Scavenging Activity; TPC, Total Polyphenol Content; CUPRAC, Cupric Reducing Antioxidant Capacity; FRAP, Ferric Reducing Antioxidant Power; ABTS, ABTS Radical Scavenging Activity

The results of relative antioxidant activity of the six investigated barley varieties, and malts produced from them are shown in Fig. 1. The 100% values for the different methods were the following in mg/100 g Ascorbic Acid Equivalent (AAE) expressed on dry matter basis (d.m.): DPPH Radical Scavenging Activity (DPPH) = 24.4, Total Polyphenol Content (TPC) = 61.9, Cupric Reducing Antioxidant Capacity (CUPRAC) = 63.3, Ferric Reducing Antioxidant Power (FRAP) = 30.5, ABTS Radical Scavenging Activity (ABTS) = 155.6. As it can be seen in Fig. 1 there are major differences between the antioxidant activities of barley varieties, which is consistent with the results obtained by Zhao et al. (2008). These difference are due to biotic and abiotic factors, which affect plant physiology and secondary metabolites such as antioxidants (Mikkelsen et al. 2015). By regulating these factors, it may be possible to effect on this property of plants. Among barleys Casanova winter cultivar has the highest DPPH (19.8 mg/100 g AAE d.m.), CUPRAC (35.3 mg/100 g AAE d.m.), FRAP (22.2 mg/100 g AAE d.m.) and ABTS (109.4 mg/100 g AAE d.m.) while Kangoo spring cultivar has the lowest values. These results can be related to the generally thicker husk of winter barley varieties. Phenolic compounds, which do contribute to antioxidant activity, are predominantly found in the outer layers of the grain, so a thicker husk can come along with a higher antioxidant activity (Naczk and Shahidi 2004; Fogarasi et al. 2015).

All the antioxidant activity levels measured by the five assays have increased during malting. This result is in accordance with the observation of Fogarasi et al. (2015)who have experienced the same in case of barley, wheat and einkorn wheat. By producing malt from barley a valuable product is made from a nutritional point of view. The highest increase during malting can be observed in TPC values, which was reported by Pejin et al. (2009) as well. The biggest increase of TPC during malting was indicated by Casanova winter cultivar, from 8.1 to 61.6 mg/100 g AAE d.m. As this method is not selective to phenolic substances, rather shows the reducing capacity of the samples (Huang et al. 2005) it cannot be declared that it goes along with the increase of phenolic content. On the other hand, there are studies, which have proven that due to enzymatic release of bound phenolic compounds of barley and easier extractability lead to higher levels of free phenolics in malt compared to barley (Carvalho et al. 2015; Dvoráková et al. 2008). In case of TPC, CUPRAC and FRAP the highest values belong to the malt made from Quench spring barley, while malt made from Kangoo spring barley shows the lowest results in general. It may be due to the malting process as the same technology was applied for all the barleys, and this technology may not have been optimal for all the samples. These conditions favored the germination of Quench barley.

### Antioxidant activity during malting

**Fig. 2:**
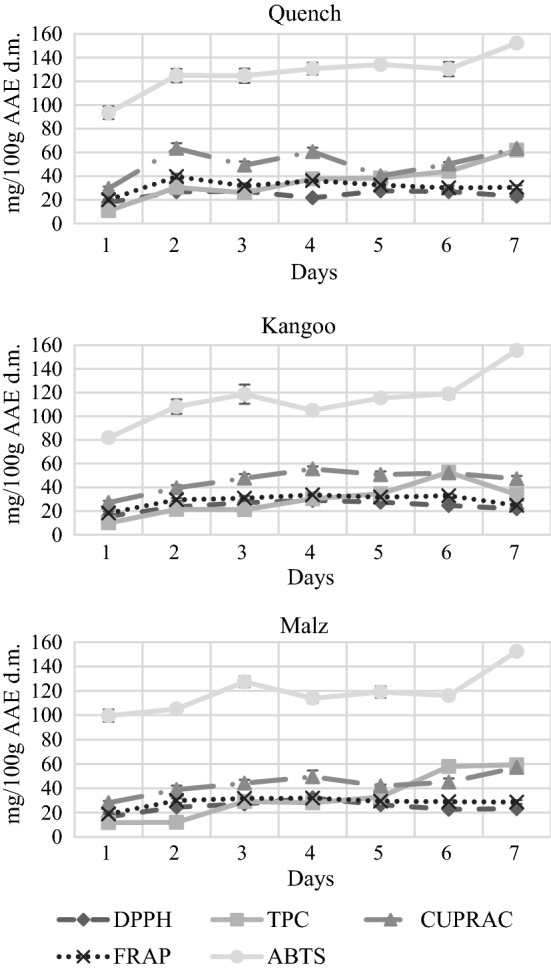
Antioxidant activity during the malting process of spring barley varieties (Quench, Kangoo, Malz). Values in mg/100 g Ascorbic Acid Equivalent related to dry matter (n = 3, mean, sd). Day 1,barley before malting; Day 2, after steeping; Day 3–6, during germination; Day 7, final malt after kilning. DPPH, DPPH Radical Scavenging Activity; TPC, Total Polyphenol Content; CUPRAC, Cupric Reducing Antioxidant Capacity; FRAP, Ferric Reducing Antioxidant Power; ABTS, ABTS Radical Scavenging Activity

**Fig. 3:**
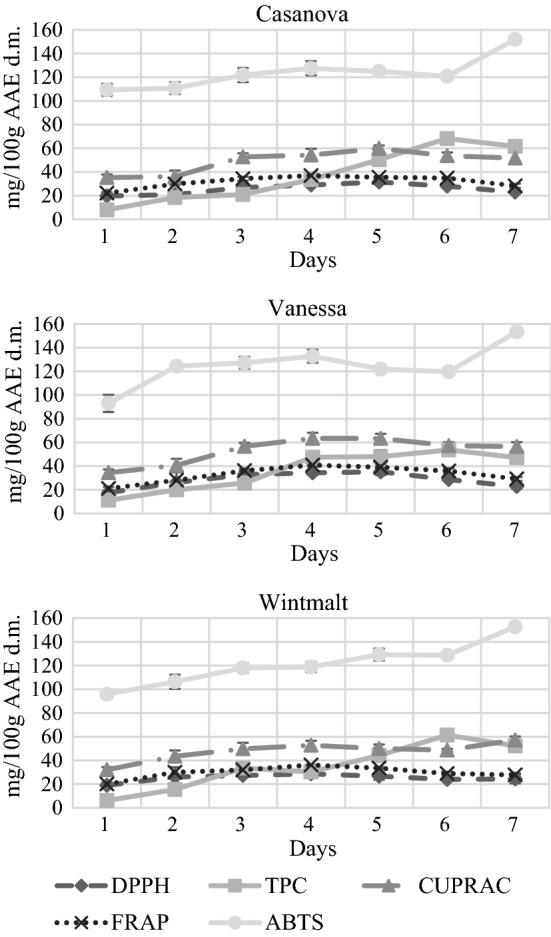
Antioxidant activity during the malting process of winter barley varieties (Casanova, Vanessa, Wintmalt). Values in mg/100 g Ascorbic Acid Equivalent related to dry matter (n = 3, mean, sd). Day 1,barley before malting; Day 2, after steeping; Day 3–6, during germination; Day 7, final malt after kilning. DPPH, DPPH Radical Scavenging Activity; TPC, Total Polyphenol Content; CUPRAC, Cupric Reducing Antioxidant Capacity; FRAP, Ferric Reducing Antioxidant Power; ABTS, ABTS Radical Scavenging Activity

In Figs. 2 and 3 the results of antioxidant activity during the malting process of spring and winter barley cultivars can be seen. As we could see on Fig. 1, the antioxidant activity measured by all the assays are higher of malts than barleys, on the other hand it does not increase continuously through the entire process. Our DPPH results are very similar to the results of Pejin et al. (2009), an increase can be observed at the end of steeping, and on the first days of germination, and after that it starts to decrease. On the other hand Lu et al. (2007) have experienced a decrease at the end of steeping by ABTS, TPC and DPPH methods with a very similar sample extraction as ours. They have reported the highest increase of DPPH during kilning, which does not agree with either our or with Pejin et al. (2009) results. This is probably due to the differences in technology and equipment as in both Peijin’s and our study a Schmidt-Seeger micromalting plant was used while the equipment of Lu is unknown. Spring and winter cultivars showed similar tendency during the whole malting process as the same biochemical processes occur during their malting. The highest increase is observed during steeping. Antioxidants are more extractable as moisture content of kernels is raised from 11 to 12% (raw barley) to 40%.

During kilning the antioxidant activity assays show different tendencies. It is maybe due to the different sensitivity of the assays to the products being formed or degraded during kilning. According to Bellmer (1978) the kilning step is regarded important for polyphenol solubilization, Leitao et al. (2012) experienced the highest increase of total phenolic content during kilning.

### Antioxidant activity during brewing

**Fig. 4:**
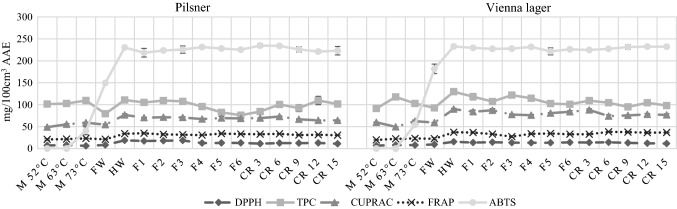
Antioxidant activity during the production of beers under laboratory circumstances. Values in mg/100 cm^3^ Ascorbic Acid Equivalent (n = 3, mean, sd). M 52 °C, after 52 °C enzymatic rest during mashing; M 63 °C, after 63 °C enzymatic rest; M 73 °C, after 73 °C enzymatic rest; SW, sweet wort; HW, hopped wort; F 1–6, days of fermentation; CR 3–15, days of chill-rest. DPPH, DPPH Radical Scavenging Activity; TPC, Total Polyphenol Content; CUPRAC, Cupric Reducing Antioxidant Capacity; FRAP, Ferric Reducing Antioxidant Power; ABTS, ABTS Radical Scavenging Activity

**Fig. 5:**
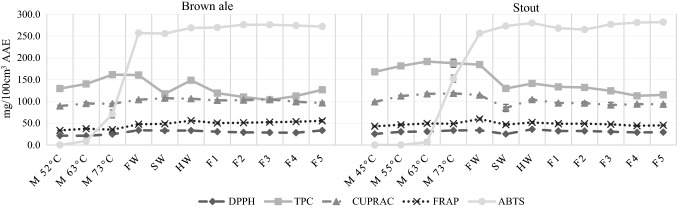
Antioxidant activity during the production of beers in pilot brewery. Values in mg/100 cm^3^ Ascorbic Acid Equivalent (n = 3, mean, sd). M 45 °C, after 45 °C enzymatic rest during mashing; M 52 °C, after 52 °C enzymatic rest; M 55 °C, after 55 °C enzymatic rest; M 63 °C, after 63 °C enzymatic rest; M 73 °C, after 73 °C enzymatic rest; FW, first wort; SW, sweet wort; HW, hopped wort; F 1–5, days of fermentation. DPPH, DPPH Radical Scavenging Activity; TPC, Total Polyphenol Content; CUPRAC, Cupric Reducing Antioxidant Capacity; FRAP, Ferric Reducing Antioxidant Power; ABTS, ABTS Radical Scavenging Activity

In Figs. 4 and 5 the results of antioxidant activity during the entire brewing process can be seen under laboratory conditions and in the pilot brewery. As can be seen, Pilsner beer has the lowest values through the entire process even though it has almost the highest original extract content among all the investigated beers, 16.51 °B, measured from its hopped wort (Table 1). Vienna lager has slightly higher antioxidant activity except for DPPH results, furthermore it has the highest original extract content, 17.28 °B of hopped wort. In general, Brown ale and Stout, that contain special malts have higher antioxidant activity, even though they have lower original extract content (Brown ale: 14.08 °B, Stout: 14.07 °B, also determined from their hopped wort) than the pale beers. From this it can be concluded that the extract content does not necessarily have an effect on antioxidant activity, much more, the malt composition affects this parameter. This result agrees with the result reported by Ditrych, Kordialik-Bogacka and Czyżowska (2016) who have found that darker beers have higher antioxidant potential than pale ones. This is mainly due to the special malts used for their production, as special malts contain more melanoidins, which contribute to the antioxidant activity (Zhao and Zhao 2012).

**Table 1:** Extract content of worts during the brewing of different types of beers

Beer type	Sample name	Extract content [°B]
Pilsner	First wort	13.68
	Hopped wort	16.51
Vienna lager	First wort	13.54
	Hopped wort	17.28
Brown ale	First wort	15.16
	Sweet wort	12.67
	Hopped wort	14.08
Stout	First wort	15.80
	Sweet wort	11.96
	Hopped wort	14.07

First wort, wort separated from the mash; Sweet wort, first wort plus wort after sparging; Hopped wort, wort after hop boiling

The TPC, CUPRAC, FRAP, DPPH values are relatively high after the first enzymatic rest at 45 °C (Stout) or 52 °C (Pilsner, Vienna lager, Brown ale). It is consistent with the result of Schwarz, Boitz and Methner (2012), who have reported the same temperature as ideal one for polyphenol release from malt, and with the results of Zhao and Zhao (2012) who have found that antioxidant activity increased the most intensive during the early stage of mashing. Until the end of the first enzymatic rest water-soluble antioxidants can go into solution, furthermore these temperatures are optimal for protease and β-glucanase enzymes of barley malt which can release antioxidants bound to cell walls, polysaccharides or proteins. By holding this temperature longer more antioxidants can be released, on the other hand the quality of the final product can be negatively affected due to excessive degradation of proteins.

The results given by ABTS assay at the early stages of the brewing process both under laboratory circumstances and in the pilot brewery were unexpected. ABTS does not show any relevant antioxidant activity until the end of the enzymatic rest at 73 °C but afterwards increases radically. In case of this assay the above elaborated theory about the importance of the protease, β-glucanase rest at the beginning of mashing is inappropriate. Compounds measured by this method can be either soluble only above 70 °C or they are formed at this higher temperature.

The wort separation using filter paper under laboratory circumstances caused an unexpected radical decrease in TPC values. Probably compounds were bound by the filter paper, which have antioxidant activity selective to TPC assay. On the other hand, ABTS and DPPH increased during wort separation. Therefore, several methods are justified to determine antioxidant-activity, because each of them determines this attribute based on another mechanism.

After first wort separation sparging is the step when the spent grain, which forms the filter layer, is washed through with hot water (in our case 78 °C water) to extract valuable components. It causes the dilution of the wort as it can be seen in Table 1. Despite of the dilution of the wort the antioxidant activity did not decrease except for TPC. In case of Stout the sparging was more intensive, the wort was diluted to a higher extent which induced the decrease of antioxidant activity. It means, that wort becomes more valuable by adequate sparging because antioxidant compounds could be further dissolved from the filter layer, which consists mostly of the husk of the kernels.

During hop boiling the antioxidant activity increased in case of all beers. During boiling Maillard reaction products are developed and the polyphenols of hops are dissolved in the wort (Pascoe et al. 2003). On the other hand, loss of antioxidant activity is experienced because polyphenols that react with proteins and form precipitation are separated with the hot trub (Kühbeck et al. 2006). The increase of antioxidant activity was higher in case of laboratory scale beers that is partly due to the better separation of the hot trub in the whirlpool of the pilot brewery than in the laboratory, and due to the more intensive evaporation during hop boiling under laboratory circumstances, the wort became more concentrated (Table 1). It seems that the different equipment used for hop boiling and the separation of hot trub can have influence on antioxidant activity. In the laboratory both hop boiling and separation of hot trub was carried out in Erlenmeyer flasks while in the pilot brewery a steam-heated wort kettle was used for hop boiling and whirlpool for the separation of hot trub.

During fermentation the antioxidant activity decreased in some cases or showed no changes as other researchers have also reported. Fantozzi et al. (1998) observed decrease while Leitao et al. (2011) have found no significant changes. In our study we have not observed difference between the evolution of antioxidant activity during main fermentations performed under different circumstances: one in fermenters of the pilot brewery and the other in a flask with airlock.

The changes during chill rest were determined only in case of beers produced in the laboratory and. no significant changes have been indicated in antioxidant activity.

## Conclusion

There are numerous studies focusing on the evolution of antioxidants during the entire brewing process but there are still gaps that need to be investigated. Brewing consists of very complex processes. The antioxidant potential of the final product depends already on the growing conditions of the raw materials used. This study revealed the differences of antioxidant activity of spring and winter barley cultivars. Despite the differences between barley varieties and despite similar trends of spring and winter cultivars during steeping and germination, kilning had different effect on antioxidant activity of the varieties. Spring and winter cultivars showed similar trends during steeping and germination, but kilning had different effect on antioxidant activity of the varieties. In the course of brewing differences between the equipment used in the laboratory and in the pilot brewery showed to have effect on antioxidant activity, especially during wort separation and hop boiling. During the mashing process the enzymatic rests at 45 °C or 52 °C are important in the release of antioxidants from malts. Together with adequate sparging and hop boiling mashing can contribute to the antioxidant activity of the final product. Sparging could be a determinative step as we can gain valuable compounds from the spent grain that would not be used in the further steps of beer production. During malting and brewing there are plenty of parameters that can be influenced by the recipe or technology to optimize the conditions for the release and retention of antioxidants. In the light of the complexity of beer production and wide choice of raw materials further research is needed to understand these processes better.

## References

[CR1] Almaguer C, Schönberge C, Gastl M, Arendt EK, Becker T (2014). Humulus lupulus–a story that begs to be told. A review. J Inst Brew.

[CR2] Apak R, Güçlü K, Özyürek M, Karademir SE (2004). Novel total antioxidant capacity index for dietary polyphenols and vitamins C and E, using their cupric ion reducing capability in the presence of neocuproine: CUPRAC method. J Agric Food Chem.

[CR3] Bellmer HG (1978) Über polyphenole und anthocyanogene in gerste und malz. Monatsschrift für Brauerei 31(3): 74–76, 78–80, 82

[CR4] Benzie IF, Strain JJ (1996). The ferric reducing ability of plasma (FRAP) as a measure of “antioxidant power”: the FRAP assay. Anal Biochem.

[CR5] Brand-Williams W, Cuvelier ME, Berset CLWT (1995). Use of a free radical method to evaluate antioxidant activity. LWT-Food Sci and Technol.

[CR6] Carvalho DO, Curto AF, Guido LF (2015). Determination of phenolic content in different barley varieties and corresponding malts by liquid chromatography-diode array detection-electrospray ionization tandem mass spectrometry. Antioxidants.

[CR7] Ditrych M, Kordialik-Bogacka E, Czyżowska A (2016). Antiradical and reducing potential of commercial beers. Czech J Food Sci.

[CR8] Dvořáková M, Douanier M, Jurková M, Kellner V, Dostálek P (2008). Comparison of antioxidant activity of barley (Hordeum vulgare L.) and malt extracts with the content of free phenolic compounds measured by high performance liquid chromatography coupled with CoulArray detector. J Inst Brew.

[CR9] Fantozzi P, Montanari L, Mancini F, Gasbarrini A, Addolorato G, Simoncini M (1998). In vitroantioxidant capacity from wort to beer. LWT-Food Sci Technol.

[CR10] Fogarasi AL, Kun S, Tankó G, Stefanovits-Bányai É, Hegyesné-Vecseri B (2015). A comparative assessment of antioxidant properties, total phenolic content of einkorn, wheat, barley and their malts. Food Chem.

[CR11] Fumi MD, Galli R, Lambri M, Donadini G, De Faveri DM (2011). Effect of full-scale brewing process on polyphenols in Italian all-malt and maize adjunct lager beers. J Food Compos Anal.

[CR12] Graf BA, Milbury PE, Blumberg JB (2005). Flavonols, flavones, flavanones, and human health: epidemiological evidence. J Med Food.

[CR13] Huang D, Ou B, Prior RL (2005). The chemistry behind antioxidant capacity assays. J Agric Food Chem.

[CR14] Kähkönen MP, Hopia AI, Vuorela HJ, Rauha JP, Pihlaja K, Kujala TS, Heinonen M (1999). Antioxidant activity of plant extracts containing phenolic compounds. J Agric Food Chem.

[CR15] Kühbeck F, Schütz M, Thiele F, Krottenthaler M, Back W (2006). Influence of lauter turbidity and hot trub on wort composition, fermentation, and beer quality. J Am Soc Brew Chem.

[CR16] Leitao C, Marchioni E, Bergaentzlé M, Zhao M, Didierjean L, Taidi B, Ennahar S (2011). Effects of processing steps on the phenolic content and antioxidant activity of beer. J Agric Food Chem.

[CR17] Leitao C, Marchioni E, Bergaentzl M, Zhao M, Didierjean L, Miesch L (2012). Fate of polyphenols and antioxidant activity of barley throughout malting and brewing. J Cereal Sci.

[CR18] Lu J, Zhao H, Chen J, Fan W, Dong J, Kong W (2007). Evolution of phenolic compounds and antioxidant activity during malting. J Agric Food Chem.

[CR19] Martinez-Periñan E, Hernández-Artiga MP, Palacios-Santander JM, ElKaoutit M, Naranjo-Rodriguez I, Bellido-Milla D (2011). Estimation of beer stability by sulphur dioxide and polyphenol determination. Evaluation of a laccase-sonogel-carbon biosensor. Food Chem.

[CR20] Mikkelsen BL, Olsen CE, Lyngkjær MF (2015). Accumulation of secondary metabolites in healthy and diseased barley, grown under future climate levels of CO2, ozone and temperature. Phytochemistry.

[CR21] Naczk M, Shahidi F (2004). Extraction and analysis of phenolics in food. J Chromatogr A.

[CR22] Narziss L (1976). Polyphenolgehalt und polymerisationsindex von gersten und kleinmalzen. Monatsschrift für Brauwissenschaft.

[CR23] Pascoe HM, Ames JM, Chandra S (2003). Critical stages of the brewing process for changes in antioxidant activity and levels of phenolic compounds in ale. J Am Soc Brew Chem.

[CR24] Pejin J, Grujić O, Čanadanović-Brunet J, Vujić Đ, Tumbas V (2009). Investigation of phenolic acids content and antioxidant activity in malt production. J Am Soc Brew Chem.

[CR25] Re R, Pellegrini N, Proteggente A, Pannala A, Yang M, Rice-Evans C (1999). Antioxidant activity applying an improved ABTS radical cation decolorization assay. Free Radic Biol Med.

[CR26] Schwarz KJ, Boitz LI, Methner FJ (2012). Release of phenolic acids and amino acids during mashing dependent on temperature, pH, time, and raw materials. J Am Soc Brew Chem.

[CR27] Shahidi F, Zhong Y, Chandrasekara A (2012). Antioxidants and human health. Cereals and pulses: nutraceutical properties and health benefits.

[CR28] Singleton VL, Rossi JA (1965). Colorimetry of total phenolics with phosphomolybdic-phosphotungstic acid reagents. Am J Enol Vitic.

[CR29] Zhao H (2015) Effects of processing stages on the profile of phenolic compounds in beer. In: Preedy V (ed) Processing and impact on active components in food, pp 533–539

[CR30] Zhao H, Zhao M (2012). Effects of mashing on total phenolic contents and antioxidant activities of malts and worts. Int J Food Sci Technol.

[CR31] Zhao H, Fan W, Dong J, Lu J, Chen J, Shan L, Kong W (2008). Evaluation of antioxidant activities and total phenolic contents of typical malting barley varieties. Food Chem.

[CR32] Zhao H, Chen W, Lu J, Zhao M (2010). Phenolic profiles and antioxidant activities of commercial beers. Food Chem.

[CR33] Zimmermann BF, Galensa R (2007). One for all—all for one: proof of authenticity and tracing of foods with flavonoids. Eur Food Res Technol.

